# The relationship between ACL reconstruction and meniscal repair: quality of life, sports return, and meniscal failure rate—2- to 12-year follow-up

**DOI:** 10.1186/s13018-020-01878-1

**Published:** 2020-08-27

**Authors:** Juan M. Rodríguez-Roiz, Sergi Sastre-Solsona, Dragos Popescu, Jordi Montañana-Burillo, Andres Combalia-Aleu

**Affiliations:** 1 Orthopedic & Trauma Surgery Dept, Hospital Clínic, C/Villarroel 170, 08036 Barcelona, Spain; 2 CLINICA SAGRADA FAMILIA BARCELONA, c/torras i pujalt 1, 08022 Barcelona, Spain

**Keywords:** ACL, Meniscal repair, Meniscal tear, Sports return, Meniscus

## Abstract

**Background:**

Few studies have approached in a long-term follow-up of meniscal repair at an amateur level, specially studying variables as a quality of life and failure rate. The purpose of this review is to study medium to long-term clinical results in patients at amateur sports patients, that have required meniscal sutures at our center, with or without ACL reconstruction. We evaluate the objective function of the knee, as well as patients’ return to sports activities, quality of life, and the rate of failed repair and study of the possible reasons.

**Methods:**

This was an observational retrospective study. Ninety-two patients who regularly perform amateur sports activities (Tegner 4 to 7) were assessed, with a minimum follow-up period of 2 years, divided into 2 groups: group 1, isolated meniscal suture (43 cases) and group 2, associated to ACL reconstruction (49 cases). Each patient made this test in 2019: Lysholm and Tegner (validated for Spanish) before a knee injury and after surgery, motivation to return to sports activity (Likert scale with 3 items: low, regular, or high), and quality of life through SD-12 test.

**Results:**

High return to amateur sports rate (92%) was even higher in the isolated meniscal repair group in comparison to the group with associated ACL. We have not found statistically significant differences between sports return and age, gender, injured meniscus, chondral injuries, preoperative Tegner score, or motivation. No significant differences in physical or mental health fields between both groups. Meniscal repair failed in 12 patients (13%). Higher rate of failure in isolated bucket-handle tear injuries (*p* < 0.0062). No statistically significant association was found between the other variables studied.

**Conclusions:**

Good results with 92% of sports return, low rate of complications, and low retear rate, even lower when is associated with ACL reconstruction and in external meniscus repair, and high values at SF-12 between 2 groups.

## Introduction

The meniscus plays a fundamental role in knee biomechanics, acting not only as a joint shock absorber, but also have proprioceptive, lubricating, and stability functions. It is well known that after a partial meniscectomy, the incidence rate of knee osteoarthritis increases [1]. Considering this, it is understandable that meniscal repair is the gold standard treatment for damaged menisci in young patients, in repairable cases.

Traumatic meniscus injuries are often associated with anterior cruciate ligament tears, chondral lesions, and medial collateral ligament injuries, among others, which can worsen the knee’s functional prognosis. In these cases, the objective must be recover function and also the patient’s quality of life, a factor which is becoming more and more important and which few studies on this topic take into consideration [1, 2].

Thirty percent to 60% of anterior cruciate ligament (ACL) tears are associated with meniscal or chondral injuries at the time of their index reconstruction [3–5], specifically the lateral meniscus, the most common. The lateral meniscus has different structural and functional properties than the medial meniscus that explains this situation; first, a lack of attachments to the popliteal hiatus and collateral ligament and a relative greater mobility, that reduce the risk of ruptures, which is the reason this meniscus is rarely affected in a stable knee [6].

Regarding the role of the menisci as secondary stabilizers within the knee, it is well known that the clinical results of anterior cruciate ligament (ACL) reconstruction are markedly impaired by the presence of concurrent meniscal injury. The anterior drawer was not increased significantly after medial meniscectomy when the further increase in tibial anterior translation of up to 5.8 mm [7], thus highlighting the importance of the meniscus-meniscal ligament construct as a secondary joint stabilizer in synergy with the ACL.

Similarly, the meniscus acts as secondary restraints to tibial internal–external rotation. Wang and Walker [8] found that meniscectomy increased the range of rotation 5° from a mean of 18–23°, at 0.5 Nm torque. The laxity was further increased if the ligaments were excised: removal of ACL and PCL (posterior cruciate ligament) increased laxity 23°, while removal of both roles of this ligament is unclear and its influence on the overall meniscal biomechanics remains elusive. It has been reported to serve in some specimens as the primary attachment of the anterior horn of the medial meniscus and proposed to act as a restraint to anterior subluxation and excessive posterior translation when the menisci are under load [9]. Finally, it is also believed that it acts as a tie between the menisci that controls their relative positioning on the tibial plateau when the tibia rotates. Failure rates of meniscal repair are “low” (< 15%) and are lower when associated with ACL reconstruction [6]. It has been reported that ACL reconstruction provides a positive biomechanical environment for the meniscus to heal [6]. The clinical picture of this situation is controversial (10). Several studies have reported both higher and similar healing rates with concomitant ACL reconstruction [11, 12].

The most of literature about meniscal repair and ACL reconstruction is focused on the professional athlete, that is not the “day by day” patient in clinics worldwide, so maybe the results that we found in literature could not be extrapolated to the most of the cases, that situation can raise high expectations for both surgeon and patient.

The medium follow-up of most of the studies about this topic is around 2–5 years, so the long-term results, specially in amateur level, has not been studied enough, and these time periods may represent a range in which also simple partial meniscectomy usually provides acceptable results.

Today, variables as the quality of life and patient satisfaction are studied much more than 10 years ago. But about meniscal repair/ACL reconstruction, there are just a few studies that review this important outcome.

The purpose of the present study was to investigate the medium to long-term (2–12 years) clinical results of amateur sports patients, who underwent isolated meniscal repair or combined with ACL reconstruction, specifically as follows:

- Determine the rate of amateur sports return using the Tegner scale to determine the sports the patient is practicing before the injury, and the actual sport, and determine not only if the patient returns, but also if he/she returns to the same Tegner sports level. We also study if there is a difference in sports return between patients who required an isolated meniscal repair and the patients who also required an ACL reconstruction. The patient’s motivation to come back to sports practice is measured.
- Functional results measured by Lysholm score
- Quality of life and satisfaction with the treatment performed, using SF-12 test, evaluating mental, and physical scale
- Failure rate of meniscal repair and the possible reasons to explain that cases diagnosing the meniscal repair failure combining clinical and MRI results

The hypothesis is that patients in both groups (isolated meniscal repair, or with an ACL reconstruction) will have high sports return values, with higher functionality and quality of life outcomes in the patients with isolated meniscal repair than the ACL reconstruction group, but with less meniscal repair failure in this last group. Also, highly motivated patients will have a higher return to sports rates and in less time.

## Material and methods

A consecutive series of traumatic meniscal tear cases operated at our center with arthroscopic meniscal suture with or without ACL reconstruction were assessed retrospectively, between April 2007 and April 2018, a total of 380 patients.

### Inclusion criteria

1. Patients between the ages of 13 and 55 who regularly perform recreational sports activities (Tegner 4 to 7, minimum 3 times/week), with a minimum follow-up period of 2 years. Patients were divided into 2 groups: group 1 (isolated meniscal repair) and group 2 (ACL reconstruction with meniscal repair).

2) Patients were assessed through online/in-person questionnaires, filled in between April and November 2019, including the following tests: Lysholm and Tegner (validated for Spanish) before the injury and after the surgical procedure, motivation to return to sports activity (Likert scale with 3 items: low, regular, or high), quality of life (through SF-12 test), and meniscal repair failure (all cases diagnosed by a MRI with compatible symptoms, and a second surgery was required). Data about the type of meniscal injury and the type of repair was obtained from the surgical report.

3) An observational retrospective study was conducted, according to the WMA Declaration of Helsinki, and was reviewed and approved by our institutional review board (document attached).

### Exclusion criteria

1. Patients with less than 13 years or more than 55 years at the time of surgical intervention
2. Isolated ACL reconstructions or with a meniscectomy (no repair)
3. Sedentary patient or professional athlete
4. Prior surgeries on the injured knee
5. Degenerative chondral injuries (Outterbridge III or more)
6. Discoid meniscus or multiligamentous injuries
7. Postoperative follow-up of less than 1 year, patients impossible to locate or those who did not want to participate in the study
8. Patients that need meniscal root reinsertion or meniscal ramp reconstruction

Finally, 92 patients were included in the study, divided into 2 groups: group 1 corresponded to the patients that required isolated meniscal suture (43 cases), and group 2 included the cases that required meniscal suture associated with ACL reconstruction (49 cases).

- *Group 1* (isolated meniscal suture) included 43 patients, 28 men and 15 women, with an average age of 32.18 years (13–47 years), with an approximate follow-up period of 7 years (2–12.5 years). The interval between injury and surgical intervention was an average of 11.74 months (1–50 months). An average of 2 meniscal sutures (1.93) was used in each case (1 to 3) (Table 1).

- *Group 2* (ACL reconstruction plus meniscal suture) included 49 patients, 35 men and 14 women, with an average age of 29.71 years (16–54 years), with an approximate follow-up period of 6 years (2–12.5 years). The interval between injury and surgical intervention was an average of 16.48 months (2–80 months). An average of 2 meniscal sutures (1.73) was used in each case (1 to 3) (Table 1).

**Table 1:** Demographic characteristics of groups

	Group 1 (isolated meniscal repair)	Group 2 (concomitant ACL)	*P*
*N*	43	49	
Sex			0.52
Male	28 (65.12%)	35 (71.43%)	
Female	15 (34.88%)	14 (28.57%)	
Age at the time of surgery	32.19 (13–52)	29.71 (14–54)	0.16
Motivation			0.34
Low	5 (11.63%)	3 (6.12%)	
Regular	15 (34.88%)	16 (32.65%)	
High	23 (53.49%)	30 (61.22%)	
Affected site			0.92
Right	25 (58.14%)	28 (57.14%)	
Left	18 (41.86%)	21 (42.86%)	
Meniscal lesions			
Right	28 (65.12%)	38 (77.55%)	
Left	15 (34.88%)	9 (18.37%)	
Both	0 (0%)	2 (4.08%)	
Type of meniscal lesion			
Simple	33 (76.74%)	45 (91.84%)	
Bucket handle	10 (23.26%)	4 (8.16%)	
Repair technique			
All inside	36 (83.72%)	47 (95.92%)	
Both	7 (16.28%)	2 (4.08%)	
No. of sutures used			
1	9 (20.93%)	19 (38.78%)	
2–3	28 (65.12%)	24 (48.98%)	
4 or more	6 (13.95%)	6 (12.24%)	
Chondral lesions	3 (6.98%)	1 (2.04%)	0.22
Reoperation	7 (16.28%)	5 (10.12%)	< 0.001
Second surgery			
Meniscectomy	7 (100%)	4 (80%)	
Debridement	0	1 (20%)	

### Surgical technique

All patients were evaluated, as usual, by an internal medicine or anesthesiologist before the surgical procedure. The surgical procedure was performed in a supine position, under spinal anesthesia plus endovenous sedation, with the affected knee flexed and with a pneumatic tourniquet cuff on the ipsilateral thigh.

In all cases, the arthroscopic meniscal suture was performed through 2–3 conventional arthroscopic portals (anterolateral, anteromedial, and accessory anteromedial), and the chosen suture technique was the Fast-Fix type all-inside suture (Smith-Nephew) and outside-in suture specifically for body and anterior horn injuries (less usual). The length of the suture was usually 16 mm for the internal meniscus and 18 mm for the lateral meniscus. In all the cases with the internal meniscus injury, we perform a “pie-crust” of the superficial internal collateral ligament to have more space and avoid iatrogenic chondral injuries. We usually made a conscious debridement of meniscal borders before the repair and also microfractures in the medial femoral condyle to favor the meniscal healing process.

In the cases that required ACL reconstruction, the hamstring autograft was the graft of choice in our study. In all cases, we initially obtained the autograft following the traditional technique with a surgical incision near the anterior tibial tuberosity in the proximal-medial tibia. The graft was measured, and bony tunnels were drilled based on that size. An anatomical reconstruction technique was used in all cases.

The femoral tunnel was always drilled in knee hyperflexion (> 110°) using an anteromedial portal and using landmarks to select the anatomical position. In some cases, we use a lateral knee X-ray intraoperatively to confirm the position. Endobutton system (Smith-Nephew) was used in all cases for femoral fixation, and for tibial fixation, we use bioreabsorbable screws always tightened in knee semi-flexion (15°) and performing a posterior draw force. Surgical closure with stitches as usual, and we usually use 2 drainages for 24 h, one for graft donor zone and another intra-articular.

### Rehabilitation and postoperative protocol

Patients who underwent isolated meniscal repair and those who underwent meniscal repair with ACL reconstruction completed the same postoperative rehabilitation program.

The patients were discharged home 24 h after surgery. In all cases, a Don-Joy knee brace was placed maximum at 60° of flexion. For the first 2 weeks, patients make non-weight-bearing with the help of 2 crutches and begin immediate rehabilitation treatment: local anti-inflammatory treatment + progressive passive knee kinesiotherapy + isometric quadricep exercises. After 2 weeks, they were authorized to begin walking with progressive partial weight-bearing. One month after surgery, they were authorized to flex to 90° for 1–2 weeks, as well as full weight-bearing. On week 6 after surgery, the brace was maintained without flexion limitation and they began walking independently without crutches.

### Outcome measures

#### Lysholm score

The Lysholm Knee Scoring Scale has an extended use beyond evaluating outcomes of knee ligament surgery. It can also be used for meniscal tears, knee cartilage lesions, osteochondritis dissecans, traumatic knee dislocation, patellar instability, patellofemoral pain, and knee osteoarthritis.

It consists of eight items that measure pain (25 points), instability (25 points), locking (15 points), swelling (10 points), limp (5 points), stair climbing (10 points), squatting (5 points), and the need for support (5 points). Every question response has been assigned an arbitrary score on an increasing scale. The total score is the summation of each response to the eight questions and may range from 0–100. Higher scores indicate a better outcome with fewer symptoms or disability.

A study developed out by Briggs et al. showed that the Lysholm questionnaire has acceptable test-retest reliability, floor and ceiling effects, criterion validity, construct validity, and responsiveness to change [13]. Finally, the questionnaire itself is relatively easy for patients to complete and does not have a complicated scoring methodology.

### Tegner activity score

The Tegner activity scale was first described in 1985 and initially designed for physician administration after ACL and meniscal injuries. To date, the Tegner activity score has been a frequently used patient-administered activity rating system for patients with various knee disorders.

The Tegner activity scale classifies both sports and work activities into one questionnaire using an 11-level gradient. Competitive sports make up the top 3 levels (levels 10–8), competitive and recreational sports categories both appear in level 7, and “other recreational sports” make up level 6. Levels 5 through 1 combine work and sports together, and level 0 indicates sick leave or disability because of the knee condition.

### Motivation to sports return

Motivation involves the internal processes that give behavior its energy and direction. Motivation originates from a variety of sources (needs, cognitions, and emotions), and these internal processes energize behavior in multiple ways such as starting, sustaining, intensifying, focusing, and stopping it [14].

We use a unipolar Likert scale question type to measure this value. For the question, How much motivated you are to return to your sports activities? The three options were (1) low motivated, (2) more or less motivated, and (3) highly motivated.

### SF-12 health survey test

The 12-item Health Survey (SF-12) was developed as a shorter alternative to the SF-36 for use in large-scale studies, and its reliability and validity have been documented [15]. All 12 items are used to calculate the physical and mental component summary scores (PCS-12and MCS-12) by applying a scoring algorithm empirically derived from the data of a US general population survey. The SF-12 test ranges from 0 to 100, where a higher score implies a better quality of life regarding health [16, 17].

### Statistical analysis

Statistical analysis was performed using SPSS version 23.0 (IBM. New York, USA). The following descriptive variables were calculated: mean, standard deviation, median, range, frequency, and percentage. The normality of the distribution for measured outcome variables was evaluated using the Kolmogorov-Smirnov test. An independent sample *t* test was used to detect differences between preoperative and postoperative outcome scores for all outcomes measured. The alpha level of significance was set at *p* < 0.05.

## Results

In 92 cases, 7 meniscal repair failures (16.27%) occurred in group 1 (isolated meniscal repair) and 4 failures (8.16%) in group 2 (ACL reconstruction plus meniscal repair); the mean time until a tear reoccurred was 2.7 years (range, 1.3–4.4 years) and 5.0 years (range, 0.8–7.5 years), respectively. When analyzing subgroups of patients with recurring tears compared to the group of patients that did not require another surgery, those with a higher frequency of bucket-handle injuries were the ones that failed. No differences have been found regarding the presence of an ACL injury or the number of sutures used (Table 2).

**Table 2:** Comparative patient characteristics

	Group 1 (isolated meniscal repair)	Group 2 (concomitant ACL)	*P*
Duration from injury to surgery (months)	11.74 ± 11.28	16.49 ± 13.30	0.06
Lysholm score	89.35 ± 10.66	84.7 ± 16.10	0.10
SF12-PCS	52.51 ± 8.5	52.57 ± 7.01	0.97
SF12-MCS	55.83 ± 8.09	54.12 ± 7.77	0.30
Tegner scale			0.59
Preoperative	5.11 ± 1.37	6 ± 0.98	
Postoperative	5.46 ± 1.44	5.31 ± 1.43	

### Lysholm score

Group 1 (isolated meniscal suture) included 43 patients, 28 men and 15 women, with an average age of 32.18 years (13–47 years), with an approximate follow-up period of 7 years (2–12.5 years). The interval between injury and surgical intervention was an average of 11.74 months (1–50 months) (Table 1). The average Lysholm test was 89.34 (58–100), indicating excellent results (95–100) in 15 patients (35%), good results (84–94) in 20 cases (47%), fair results (65–83) in 6 cases (14%), and poor results (< 64) in only 2 cases (4.65%) (Table 1).

Group 2 (ACL reconstruction meniscal suture) included 49 patients, 35 men and 14 women, with an average age of 29.71 years (16–54 years), with an approximate follow-up period of 6 years (1–12.5 years). The interval between injury and surgical intervention was an average of 16.48 months (2–80 months). The average Lysholm test was 84.69 (15–100), indicating excellent results (95–100) in 10 patients (20.40%), good results (84–94) in 27 cases (55.1%), fair results (65–83) in 8 cases (16.32%), and poor results (< 64) in only 4 cases (8.16%) (Table 3).

**Table 3:** Comparative meniscal failed suture vs successful reconstruction

	Failed meniscal suture	Successful meniscal repair	*P*
Sex			0.19
Male	10 (83.33%)	53 (66.25%)	
Female	2 (16.67%)	27 (33.75%)	
Age	29.92	31.01	0.67
Motivation			0.52
Low	3 (25%)	5 (6.25%)	
Regular	2 (16.67%)	29 (36.25%)	
High	7 (58.33%)	46 (57.5%)	
Affected knee			0.95
Right	7 (41.67%)	46 (57.5%)	
Left	5 (58.33%)	34 (42.5%)	
Injured meniscus			0.67
Internal	9 (75%)	57 (71.25%)	
External	3 (25%)	21 (26.25%)	
Both		2 (2.5%)	
Type of meniscal lesion			**0**.**0062**
Simple	7 (58.33%)	71 (88.75%)	
Bucket handle	5 (41.67%)	9 (11.25%)	
Repair technique			
All inside	9 (75%)	74 (92.5%)	0.057
Both	3 (25%)	6 (7.5%)	0.29
No. sutures used			0.68
1	2 (16.67%)	26 (32.5%)	
2–3	8 (66.67%)	44 (55%)	
4 or >	2 (16.67%)	10 (12.5%)	
Chondral lesions	1 (8.33%)	3 (3.75%)	0.49
Lysholm	83.92	87.31	0.51
SF12-MCS	55.33	54.86	0.81
SF12-PCS	50.5	52.85	0.4
Tegner			
Preop	5.33	5.62	0.48
Postop	4.08	5.56	**0**.**001**
Duration from injury to surgery (months)	10.92	14.77	0.12
Group			
Combinated acl	5	44	0.38
Isolated	7	36	0.38

None statistically significant differences were found between both groups (*p* = 0.10).

### Tegner activity score

In group 1, with an average follow-up period of 7 years (2–12.5 years), patients presented a discrete increase in their baseline Tegner score from 5.11 (± 1.37) to 5.46 (± 1.44). Of the 43 patients, only 2 did not return to exercise.

In group 2, with an average follow-up period of 6 years (1–12.5 years), patients presented a discrete decrease in their baseline Tegner score from 6 (± 0.98) to 5.31 (± 1.43). Out of 49 patients, 5 did not return to exercise.

No statistically significant differences were found regarding the pre- and postoperative Tegner score in either group (*p* = 0.59), or regarding giving up amateur sports activities (Table 3).

### SF-12 health survey test

In group 1, all patients except three declared they were very satisfied with the postoperative outcome in both physical and mental health fields (ranging from 30.8 to 63.5 and 32 to 68, respectively) and further indicated that they would practice the same procedure under the same circumstances (Table 3).

In group 2, all patients except six were very satisfied with the postoperative outcome in both physical and mental health fields (ranging from 26.93 to 63 and 26 to 68, respectively) (Table 3).

No significant differences were found between both groups regarding physical or mental health fields (Table 3).

### Complications and re-interventions

The principal cause for re-intervention in our series was due to meniscal suture failure, and one case of local debridement (donor zone) with no other relevant complication requiring further surgery (septic arthritis, ACL re-rupture, etc.). We considered meniscal suture failure in patients with persistent or recurrent symptoms attributable to meniscal injury worthy of another surgical intervention. In all cases, the clinical diagnosis was confirmed with an MRI and a partial arthroscopic meniscectomy was subsequently performed on the failed repair area. In group 1, out of 43 patients, the meniscal suture failed in 7 cases (16.28%); 6 cases of medial meniscus repair (2 bucket-handle tears) and 1 case of lateral meniscus repair. There were 4 cases of all-inside suture, and 3 cases of hybrid technique (all-inside and inside-out). In 4 cases, 2–3 sutures had been used; in 2 cases, 4 sutures or more; and in 1 case, 1 suture. The average time between both surgeries was 3.17 years (8 months–11 years), with an average age group of 33 years (17–47 years).

In group 2, out of 49 cases, the meniscal suture failed in 5 cases (10.20%); 2 cases of a lateral meniscus repair and 3 cases of medial meniscus repair (1 bucket-handle tear). In all cases, an all-inside suture had been performed: in 1 case with 1 suture and in 4 cases with 2 to 3 sutures. The average time between both surgeries was 3.6 years (1–7 years), with an average age group of 26 years (18–32 years).

All cases of meniscal suture failure required a partial meniscectomy and were reevaluated 2 years later with the same initial tests.

Cumulative incidence values for failure were determined along with their 95% CIs for the various demographic predictors analyzed during Cox proportional hazards regression (Table 2). No single variable was found to predict increased failure incidence over time.

## Discussion

### Meniscal repair outcomes

Good clinical results after meniscal repair with or without ACL reconstruction were obtained, with a low suture failure rate and few complications. We found a statistically significant difference (*p* < 0.001) in meniscal repair failure between groups. In group 1 (isolated meniscal repair), 7 of 43 repairs fail and required a partial meniscetomy; in group 2 (ACL reconstruction associated), just 4 of 49; therefore, ACL reconstruction must have a “protective influence” on a meniscal repair. A systematic review by Paxton et al. [18] showed an overall reoperation rate of 24% after meniscal repair compared with 14% when performed in conjunction with ACL reconstruction, and this relation was maintained even when analyzed by specific repair methods and devices.

In a study of reoperations after a meniscal repair, with and without concomitant ACL reconstruction, Wasserstein et al. [19] concluded that meniscal repair performed in conjunction with ACL reconstruction carries a 7% absolute and 42% relative risk reduction of reoperation after 2 years compared with isolated meniscal repair.

A potential explanation for better results when a meniscal repair is associated to ACL reconstruction, both in literature and in our study, could be the blood and bone marrow cells that are liberated after drilling bony tunnels, the biomechanical stability that ACL reconstruction gives to the knee explains the results, the relatively limited patient activity, and maybe the less aggressive rehabilitation after combined procedures. Also, we found a higher failure for an internal meniscus repair (9 failures in 66 repairs) than external (3 fails of 24 repairs), no significant difference, but can be explained by the stability of meniscus, and external meniscus has higher mobility upon the tibial plateau so it has more resistance to shear forces [20, 21].

The outside-in suture has traditionally been considered the gold standard of meniscal repair, although recent studies have found similar results with all-inside suture techniques, and, specifically in bucket-handle tears, no clinically significant differences have been found in the medium term between all-inside or inside-out meniscal sutures [10, 11]. Biomechanical studies show the same resistance to load-bearing in both repair techniques [21, 22].

The literature reports an incidence of meniscal retears ranging from 20% to almost 40%. The risk factors for meniscal retears were the size (length of the tear), the presence of a complete tear, and a positive pivot-shift test finding (residual instability) [23].

In this study, the meniscal repair failed in 12 patients (13%). We observed a higher rate of suture failure in isolated bucket-handle tear injuries in comparison to other types of meniscal injuries, which is a statistically significant difference (*p* < 0.0062). We didnt found clinically significant differences with regard to recurring tears when comparing all-inside technique with cases using the hybrid technique (outside-in + all-inside). A statistically significant association between the other variables studied and meniscal repair failure was not found: age, gender, knee laterality, injured meniscus, number of sutures used, preoperative Tegner score, time between injury, and surgical intervention or chondral injuries (Table 2).

Traditionally, many experts consider age to be a key factor, ruling out the possibility of meniscal repair in patients over 50 years of age. A recent meta-analysis did not find differences between repair and recurring meniscal tear rates in patients over 40 years of age in comparison to younger patients [24]. We agree with recent literature; we have not observed statistically significant differences between the patient’s age and the failure rate of meniscal repair (Table 2).

The type of repair is also important, with several studies proving higher resistance and lower recurring tear rates in vertical sutures compared to horizontal sutures [2, 8, 13]. Indeed, perhaps this is one of the reasons behind the low recurring tear rate in our series, since in almost all cases, we tended to use vertical sutures.

In all the patients who presented a meniscal suture failure that required partial meniscectomy, we observed a statistically significant (*p* < 0.001) logical decrease in the Tegner scale (with regard to the initial preoperative score), and an equally lower return rate to sports activities. Nevertheless, when assessing medium to long term, we observed in our series that from functional (Lysholm) and quality-of-life (SF-12) point of view, patients in this group ended up with very similar results to those with a successful meniscal suture. For this reason, we consider selective partial meniscectomy to be a valid option to be considered in cases of failed suture.

### Sports return

Returning to sports activities is one of the fundamental objectives of meniscal reconstructions, with or without ACL reconstruction. The literature describes a very high percentage of patients that return to sports activities satisfactorily [25, 26]. Our series also revealed a high return rate (92%), which was even higher in the group 1 (isolated meniscal repair) in comparison to group 2 (ACL associated) (Table 4), which can be explained by the further complexity of ACL injuries, but no statistical diference for this result (*p* = 0.38).

**Table 4:** Comparative meniscal sports return

	Abandoned sport	Sports return	*P*
Number of cases	7 (7.6%)	85 (92.4%)	
Sex			
Male	4	59	
Female	3	26	
Age	31,85	30,78	
Motivation			
Low	1	7	
Regular	4	27	
High	2	51	
Affected knee			
Right	7	46	
Left	0	39	
Injured meniscus			
Internal	6	60	
External	1	23	
Both	0	2	
Type of meniscal lesion			
Simple	4	74	
Bucket handle	3	11	
Repair technique			
All inside	6	77	
Both	1	8	
No. Sutures used			0.68
1	2 (16.67%)	26 (32.5%)	
2–3	2 (16.67%)	44 (55%)	
4 or >		10 (12.5%)	
Chondral lesions	1 (8.33%)	3 (3.75%)	0.49
Lysholm	91.5	85	0.51
SF12-MCS	54.65	58.14	< 0.001
SF12-PCS	52.72	50.43	< 0.001
Tegner			
Preop	5.33	5.62	0.48
Postop	4.08	5.56	**0**.**001**
Duration from injury to surgery (months)	10.92	14.77	0.12
Group			
Combinated ACL	5	44	0.38
Isolated	7	36	0.38

Known factors affecting sports return include graft type, patient age, baseline activity level, sport type, and athletic experience within the sport.

We have not found statistically significant differences between returning to sports activities and age, gender, injured meniscus, Lysholm, chondral injuries, preoperative Tegner score, or motivation to return to sports activities (Table 4).

As a curiosity, we find a higher Lysholm medium score in patients who abandoned sports (91.5) than who returned (85); the difference is not statistically significant (Table 4, *p* = 0.51). This shows that sports return is somewhat complex and needs more than just a good functional knee, motivation, and other psychosocial factors that made an important influence [27].

Preop and postop Tegner score with a small difference in both groups and no statistically significant difference (*p* = 0.59) (Table 3), Tegner activity scores decrease specially in group 2 (from 6 ± 0.98 to 5.31 ± 1.43) for reasons unrelated to the potential function of the knee (we obtained high Lysholm scores postop), that is, by factors unrelated to knee function such as a different social setting in midlife with less time for amateur sports and more focus on family and career.

### Functional knee results

We use Lysholm test to measure objectively the function of the knee. Group 1 (isolated meniscal repair) have higher medium Lysholm (89.34) than group 2 (meniscal repair with ACL reconstruction) with 84.69 (Table 3), but the small diference was not statistically significant (*p* = 0.10). The difference can be easily answered because the association between ACL tear and meniscus usually means a higher joint damage and a more complex surgery and rehabilitation process, than an isolated meniscal tear.

We obtain similar Lysholm scores than literature. Zheng et al. [28] reported Lysholm values of 87.7 + 8.5 with a medium follow-up of 2 years in ACL reconstruction with autograft. Shirish et al. [29] found a medium Lysholm score of 91.4 at a 2-year follow-up of meniscal repair and ACL reconstruction follows.

### Quality of life

Our study revealed no statistically significant difference both physical and mental areas from SF-12 (*p* = 0.97 and *p* = 0.30) between the group of patients with isolated meniscal suture or ACL reconstruction when it comes to the quality of life (SF-12) with similar results, with little influence from chondral injuries also, although it must be noted that our study only included Outterbridge stage 2 chondral injuries at the most (Table 2). Chondral injuries at the time of ACL reconstruction have more medium-term impact on the quality of life (quantified by SF-12) and functional recovery (WOMAC) than meniscal injuries; subsequently, patients with chondral injuries also have worse results for these variables [6, 17, 18, 30].

Fuch et al. found high outcomes in the quality of life in isolated meniscal repair, with a follow-up of 3 years, measured by KOOS QOL, with a medium score of 81.8 ± 12.1 [31].

In a recent retrospective study by Cinque et al. [32], in 85 patients with ACL reconstruction with or without meniscal repair, they found similar outcomes in quality of life than our study, using SF-12 they found in physical components medium value of 52.8, and in mental components, they found 53.2. And also they did not found a statistically significant difference between the ages and quality of life outcomes.

### Motivation for sports return

The motivation for sports return is fundamental in the rehabilitation of the knee injuries, because it is a hard process, both physically and mentally, so if the patient is “goal-oriented” to come back to sports, it is pretty sure this gonna boost the process. The problem is that maybe highly motivated athletes can push too further the process and maybe early overload the knee and potentially damage the meniscal repair. We did not find a statistically significant difference between motivation and meniscal successful repair or failure (*p* = 0.34) (Table 2).

Fifty-one of the 53 highly motivated patients return to sports, but 7 of 8 low motivated also return. We did not found either a statistically significant difference (*p* = 0.18)

Brewer et al. [33] prospectively examined the relationship between psychological factors and rehabilitation outcomes after ACL reconstruction in 95 patients. Self-motivation, athletic identity, and psychological distress were significant preoperative predictors of objective outcomes such as knee laxity. However, postoperative rehabilitation adherence did not affect rehabilitation outcomes.

Nwachukwu et al. [34] in a recent systematic review find that the ideal psychological measure of sports return should consider factors including willingness, motivation, and fear of returning both preoperatively and postoperatively.

More studies are necessary to establish the validity and general usability of such questionnaires in patient populations of different sporting activity levels

### Limitations

The limitations of our study need to be acknowledged. First, the strict inclusion criteria meant that the cohort sizes were fairly small. Larger sample sizes would be required to match patients based on additional criteria, such as the number of sutures.

The study was retrospective in nature, and thus, the analysis was limited to the data available in the medical record database, and with a potential for selection bias. Second, we define a meniscal successful repair as the absence of meniscal symptoms or a normal MRI, but we do not have a second-look arthroscopy confirmation; this was only performed in the cases than repair failed.

Third, we do not use objective probes (as KT-1000, etc.) to test the knee stability, specially after ACL reconstruction, we just use Lysholm as the indicator for instability. We assume that sports return and absence of symptoms is good enough, but studies with a larger sample of amateur sports players and more objective tests will be needed to confirm our results. Another weakness of the study is the lack of radiographic evaluations that might have correlated with the patient-reported outcome scores.

Despite these limitations, our study focused specifically on the isolated repair of meniscal tears in ACL-intact knees versus repair with concomitant ACL reconstruction at an amateur level.

## Conclusions

In our study, patients that required meniscal suture presented good clinical progress, both from the point of view of returning to sports activities, quality of life (measured by SF-12), and the functional condition of the knee in the medium and long term. The failure rate of the meniscal suture is relatively low (12% in our study), and is even lower when associated to ACL reconstruction, so this must have a protective function from a mechanic, and also in a biological way, we found higher meniscal repair failure in isolated internal meniscus repair than external meniscus, specially in bucket-handle injuries. Age and time from injury until surgery does not seem to influence in meniscal repair failure.

## Data Availability

Not applicable

## Abbreviations

ACL: Anterior cruciate ligament
